# Relationship between body image disturbance and incidence of depression: the SUN prospective cohort

**DOI:** 10.1186/1471-2458-9-1

**Published:** 2009-01-02

**Authors:** Adriano Marçal Pimenta, Almudena Sánchez-Villegas, Maira Bes-Rastrollo, Celeste Nicole López, Miguel Ángel Martínez-González

**Affiliations:** 1 Department of Maternal and Child Nursing and Public Health, Nursing School, Universidade Federal de Minas Gerais (UFMG), Avenida Alfredo Balena, 190. Belo Horizonte, Minas Gerais, CEP 30130-100, Brazil; 2 Department of Preventive Medicine and Public Health, Medical School, Clínica Universitaria/Universidad de Navarra (UNAV), Inrunlarrea, 1. Pamplona, Navarra, E 31080, Spain; 3 Department of Clinical Sciences, Medical School, Universidad de Las Palmas de Gran Canaria (ULPGC), PO BOX: 550, Las Palmas, Las Palmas de Gran Canaria, CP. 35080, Spain; 4 Harvard Medical School, Harvard University, 25 Shattuck Street, Boston, Massachusetts, 02115, USA

## Abstract

**Background:**

Body image disturbance is an increasing problem in Western societies and is associated with a number of mental health outcomes including anorexia, bulimia, body dysmorphia, and depression. The aim of this study was to assess the association between body image disturbance and the incidence of depression.

**Methods:**

This study included 10,286 participants from a dynamic prospective cohort of Spanish university graduates, who were followed-up for a median period of 4.2 years (Seguimiento Universidad de Navarra – the SUN study). The key characteristic of the study is the permanently open recruitment that started in 1999. The baseline questionnaire included information about body mass index (BMI) and the nine figure schemes that were used to assess body size perception. These variables were grouped according to recommended classifications and the difference between BMI and body size perception was considered as a proxy of body image disturbance. A subject was classified as an incident case of depression if he/she was initially free of depression and reported a physician-made diagnosis of depression and/or the use of antidepressant medication in at least one of the follow-up questionnaires. The association between body image disturbance and the incidence of depression was estimated by calculating the multivariable adjusted Odds Ratio (OR) and its 95% Confidence Interval (95% CI), using logistic regression models.

**Results:**

The cumulative incidence of depression during follow-up in the cohort was 4.8%. Men who underestimated their body size had a high percentage of overweight and obesity (50.1% and 12.6%, respectively), whereas women who overestimated their body size had a high percentage of underweight (87.6%). The underestimation exhibited a negative association with the incidence of depression among women (OR: 0.72, 95% CI: 0.54 – 0.95), but this effect disappeared after adjusting for possible confounding variables. The proportion of participants who correctly perceived their body size was high (53.3%) and gross misperception was seldom found, with most cases selecting only one silhouette below (42.7%) or above (2.6%) their actual BMI.

**Conclusion:**

We found no association between body image disturbance and subsequent depression in a cohort of university graduates in Spain.

## Background

Body image disturbance is defined as a distortion of perception, behavior, or cognition related to weight or shape [1], and it is becoming a common clinical disorder [2-7].

Aesthetic standards typical of Western cultures, based on the stereotype of a lean body for women and a muscular body for men, are considered possible determinants of body image disturbance [8,9]. While most women wish to lose weight [10] even when their anthropometric parameters were normal, a significant number of men have shown muscle dissatisfaction [11].

Some reports in the scientific literature have found an association between body image disturbance and a number of mental health outcomes including depression [12-17], anorexia [12-14], bulimia [12-14], and body dysmorphia [16].

Currently, depression is a major public health problem. There is an estimated 150 million people around the world who suffer from this disease, and it is the first and the fourth cause of morbidity among women and men, respectively [18].

Recent data from the European Community suggest that depression is responsible for 6.2% of all cases of disability-adjusted life years, making it the third cause of morbidity in this continent, surpassed only ischemic heart disease and stroke [19].

In Spain, the European Study of the Epidemiology of Mental Disorders (ESEMED) showed that depression was the most common mental disorder in the country's population, with a prevalence of 3.9% [20].

Depression has a significant socioeconomic impact on a society, because it can lead to absence from work. Furthermore, depression is strongly related to suicide, which is also a major health problem worldwide [18].

Thus, research of factors associated with depression is important in order to reduce the magnitude and consequences of this disease.

Results of some studies have shown that body image disturbance is associated with depression [8,10,16,17]. Other researchers suggest that depression predisposes to body image disturbance [12,13,15]. However, these previous studies had a cross sectional design, which does not assure the temporal sequence of the association investigated.

Therefore, the aim of our study was to assess the association between body image disturbance and incidence of depression in a large prospective cohort of university graduates in Spain.

## Methods

### Subjects

The SUN project (Seguimiento Universidad de Navarra) is a multi-purpose, dynamic cohort conducted in Spain, consisting of exclusively university graduates as participants. The recruitment of participants began in December 1999 and it is permanently open. Information is collected using self-administered questionnaires sent by postal mail every two years. The objectives and methods of this project have been detailed previously [21].

As of February 2008, data from 19,057 participants were coded and prepared for analysis. All participants who complete a baseline assessment (Q_0) before May 2005 were eligible for these analyses (n = 15,502). Among them, 1,852 did not answer any of the follow-up questionnaires; after five mailings, they were considered lost to follow-up. We retained 13,650 participants. Among them, 4,328 were recruited more than 6 years ago and we had data from the baseline (Q_0), the 2-year (Q_2), the 4-year (Q_4), and the 6-year (Q_6) follow-up questionnaires. The number of participants retained for the 4-year follow-up (they returned Q_2 and Q_4) and for the 2-year follow-up (they returned Q_2) were 4,265 and 5,057, respectively. The overall follow-up rate was approximately 89%.

Participants who had some of the following characteristics were excluded from the analysis (n = 3,364): subjects who reported the use of antidepressants or those with a self-reported physician-made diagnosis of depression at baseline (n = 1,565), those who reported extremely low or high values for total energy intake, because they were more likely to have failed to properly complete the questionnaire (less than 800 Kcal/day in men and 500 Kcal/day in women or more than 4000 Kcal/day in men and 3500 Kcal/day in women) (n = 1,184), we also excluded those without information on body size perception (n = 582), and pregnant women at baseline (n = 33). Finally, data from 10,286 participants remained available for the analysis.

The study was approved by the Human Research Ethical Committee at the University of Navarra. Voluntary completion of the first questionnaire was considered to imply informed consent.

### Exposure assessment

The information about self-reported BMI was collected in the baseline questionnaire (Q_0) and grouped into four categories using the cut-off points established by the WHO: BMI <= 19.99 kg/m^2 ^for underweight subjects, 20.00 kg/m^2 ^<= BMI <= 24.99 kg/m^2 ^for normal weight subjects, 25.00 kg/m^2 ^<= BMI <= 29.99 kg/m^2 ^for overweight subjects and BMI >= 30.00 kg/m^2 ^for obese subjects [22]. The self-reported BMI by participants of the SUN study was validated in a specific study exhibiting a high degree of validity (mean error = 1.4%) [23].

**Figure 1:**
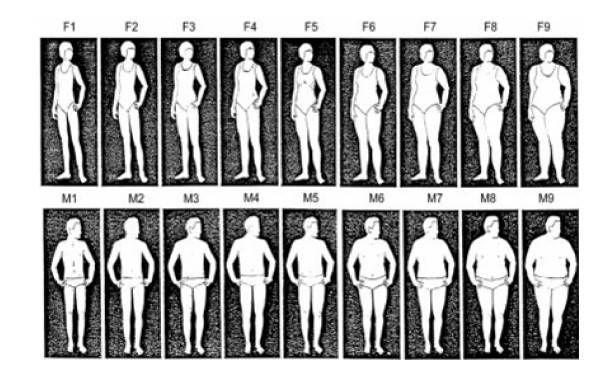
**Options presented to select their silhouettes**. F1/M1 – F3/M3 = underweight (BMI <= 19.99 kg/m^2^); F4/M4 – F5/M5 = normal weight (20.00 kg/m^2 ^<= BMI <= 24.99 kg/m^2^); F6/M6 – F7/M7 = overweight (25.00 kg/m^2 ^<= BMI <= 29.99 kg/m^2^); F8/M8 – F9/M9 = obesity (BMI >= 30.00 kg/m^2^).

Body size perception was also assessed in the baseline questionnaire (Q_0) with the nine figure scheme (Figure 1), which was validated by Stunkard [24]. Participants were asked to select which of the nine figures most closely represent their body size. After this, they were grouped into four categories following the recommended classification: pictures 1, 2 and 3 as equivalent to underweight (BMI <= 19.99 kg/m^2^), pictures 4 and 5 to represent normal weight (20.00 kg/m^2 ^<= BMI <= 24.99 kg/m^2^), pictures 6 and 7 representing overweight (25.00 kg/m^2 ^<= BMI <= 29.99 kg/m^2^) and pictures 8 and 9 representing obesity (BMI >= 30.00 kg/m^2^) [25].

The comparison between the categories of BMI and body size perception was conducted and the differences were considered as a proxy of body image disturbance. We classified individuals as overestimating their body size when they classified themselves into a higher body size category than their actual BMI. We classified individuals as underestimating their body size when they classified themselves into a lower body size category than their actual BMI.

### Covariate assessment

The baseline questionnaire (Q_0) included 554 questions about a wide array of characteristics: socio-demographic (e.g. sex, age and marital status), anthropometric (e.g. weight and height, weight gain in the past five years), lifestyle and health-related habits (e.g. smoking status, physical activity), diet (e.g. energy and alcohol intakes), employment status (e.g. employed/unemployed and working hours/week), obstetric history for women (e.g. pregnancy) and medical history (e.g. prevalence of chronic diseases and medication use). Total energy and alcohol intakes were ascertained through a semi-quantitative food frequency questionnaire (136 food items) previously validated in Spain [26]. Physical activity was collected through a validated questionnaire that included information about 17 activities such as walking, running, cycling, swimming, judo, soccer, skiing or sailing. To quantify the volume of activity during leisure time, an activity metabolic equivalent (MET) index was computed by assigning a multiple of resting metabolic rate (MET score) to each activity [27], and the time spent in each of the activities was multiplied by the MET score specific to each activity, and then summed the overall activities obtaining a value of overall weekly MET-hours. Leisure time physical activity estimated with the questionnaire was previously validated by our group using a tri-axial accelerometer as the gold standard. Physical activity during leisure time (estimated as MET-h/week) derived from the questionnaire moderately correlated with Kcal/day assessed through the accelerometer (Spearman's rho = 0.507, 95% CI: 0.232 – 0.707, p < 0.001) [28].

Participants were classified as having cardiovascular disease at baseline or at follow-up if they reported at least one of the following conditions: myocardial infarction, stroke, atrial fibrillation, paroxysmal tachycardia, coronary artery bypass grafting or other revascularization procedures, heart failure, aortic aneurism, pulmonary embolism, or peripheral venous thrombosis. Information regarding cancer both at baseline and during follow-up was also collected.

### Outcome assessment

Any participant, initially free of depression and of antidepressant treatment, who positively responded to the following question in Q_2, Q_4 or Q_6: "Have you ever been diagnosed of depression by a health professional?," was classified as an incident case of depression. A self-report of a physician-made diagnosis of depression has been validated in our cohort using the Structured Clinical Interview for DSM-IV (SCID-I) as gold standard. Sixty two subjects with a self-reported physician-made diagnosis of depression and 42 subjects without the diagnosis were included in the validation study. The percentage of confirmed depression was 74.2%; 95% CI: 63.3 – 85.1. The percentage of confirmed non-depression was 81.1%; 95% CI: 69.1 – 92.9 (submitted article).

### Statistical analysis

The analysis was stratified by sex. Non-conditional logistic regression models were fit to assess the relationship between body image disturbance and incidence of depression in our cohort. Odds Ratios (OR) and their 95% Confidence Intervals (CI) were calculated taking as the reference category those subjects who had agreement between the body size perception and self-reported BMI.

Potential confounders included in the multivariate models were: age (continuous), working hours (< 35 hours/week, >= 35 hours/week, unemployed), weight gain in the past five years (none, lost, gain), marital status (unmarried, married, other), smoking (never, past smoker and current smoker), presence of any severe disease at baseline (cardiovascular and cancer), presence of any severe disease at follow-up (cardiovascular and cancer), alcohol intake (g/day, continuous), total energy intake (Kcal/day, continuous) physical activity during leisure time (weekly MET-hours, continuous) and pregnancy at follow-up.

All p values presented are 2-tailed; p < 0.05 was considered statistically significant.

## Results

The mean follow-up of participants was 4.4 years (median = 4.2 years). Three hundred thirty eight participants initially free of depression reported a medical diagnosis of depression during this period (95 men and 243 women). In addition, there were 160 participants with no medical diagnosis of depression, but who reported initiation of antidepressants (67 men and 93 women). Therefore, the overall cumulative incidence of depression in this population was 4.8% during the follow up period.

We show in table 1 some characteristics of participants and non-participants of this study. The non-participants were older, consumed more energy, and worked less hours/week than participants. The non-participants also had a higher proportion of women, ex-smokers, overweight, and obesity than participants.

**Table 1:** Characteristics of participants and non-participants in the SUN study.

**Characteristics**	**Participants** **(n = 10,286)**	**Non-participants** **(n = 8,771)**
Incidence of depression (n, %)	498 (4.8)	105 (1.2)
Age [years] (mean, SD)^a^	37.7 (11.9)	39.2 (12.6)
Sex (n, %)^a^		
Male	4368 (42.5)	3115 (35.5)
Female	5918 (57.5)	5656 (64.5)
Body mass index [Kg/m^2^] (n, %)^a^		
Underweight	1469 (14.3)	1370 (15.6)
Overweight	2541 (24.7)	2273 (25.9)
Obese	452 (4.4)	461 (5.3)
Weight change in the past five years [Kg] (n, %)		
Lost	2466 (24.0)	2128 (24.3)
Gain	4849 (47.1)	4053 (46.2)
Marital status (n, %)^a^		
Unmarried	4597 (45.0)	3727 (42.9)
Married	5246 (51.4)	4423 (50.9)
Other	368 (3.6)	539 (6.2)
Body image (n, %)		
Underestimated	4542 (44.2)	3474 (44.9)
Overestimated	265 (2.6)	230 (3.0)

Standard deviation (SD), ^a ^Statistical significant difference.

Table 2 shows the main characteristics of participants according to the categories of the variable 'body image disturbance'. Among men, those who overestimated their body size showed higher frequency of body weight gain in the past five years, and more of them were ex-smokers compared to those who underestimated or correctly perceived their body size. The men who overestimated their body size also were older and had lower average physical activity during leisure time than those who underestimated or correctly perceived their body size. Furthermore, among men who underestimated their body size there was a higher proportion of overweight and obesity than those who overestimated or correctly perceived their body size.

**Table 2:** Characteristics of participants in the SUN study according to sex.

	**Sex**					
	
	**Male**			**Female**		
	
	**Body image disturbance**			**Body image disturbance**		
	
	**None** **n = 1,875**	**Under estimated** **n = 2,397**	**Over estimated** **n = 96**	**None** **n = 3,604**	**Under estimated** **n = 2,145**	**Over estimated** **n = 169**
Depression (n, %)	78 (4.2)	82 (3.5)	1 (1.0)	218 (6.0)	108 (5.0)	10 (5.9)
Age [years] (mean, SD)^a^	42.2 (13.0)	41.7 (12.4)	47.4 (14.8)	34.1 (10.1)	35.5 (10.4)	31.2 (9.1)
Body mass index [Kg/m^2^] (n, %)^a^						
Underweight	58 (3.1)	0 (0.0)	4 (4.2)	1,259 (34.9)	0 (0.0)	148 (87.6)
Overweight	682 (36.4)	1202 (50.1)	4 (4.2)	124 (3.4)	529 (24.7)	0 (0.0)
Obese	35 (1.9)	302 (12.6)	0 (0.0)	14 (0.4)	101 (4.7)	0 (0.0)
Weight change in the past five years [Kg] (n, %)^b^						
Lost	330 (17.6)	460 (19.2)	13 (13.5)	999 (27.7)	608 (28.3)	56 (33.1)
Gain	1,064 (56.7)	1,218 (50.8)	62 (64.6)	1,532 (42.5)	905 (42.2)	68 (40.2)
Marital status (n; %)^c^						
Unmarried	634 (34.0)	804 (33.8)	29 (30.5)	1,968 (55.0)	1,055 (49.6)	107 (64.1)
Married	1,171 (62.9)	1,495 (62.8)	66 (69.5)	1,490 (41.6)	972 (45.7)	52 (31.1)
Other	58 (3.1)	81 (3.4)	0 (0.0)	122 (3.4)	99 (4.7)	8 (4.8)
Smoking (n, %)^b^						
Current smoker	373 (20.6)	469 (20.3)	17 (17.9)	911 (26.1)	485 (23.3)	41 (25.2)
Past smoker	633 (34.9)	772 (33.3)	38 (40.0)	833 (23.9)	535 (25.7)	36 (22.1)
Physical activity [METs-h/week] (mean, SD)^b^	26.8 (23.7)	29.6 (27.0)	21.5 (19.6)	21.7 (18.6)	21.6 (18.0)	20.3 (15.9)
Energy intake [Kcal/day] (mean, SD)	2,448.9 (668.0)	2,462.5 (663.2)	2,375.2 (686.1)	2,320.4 (573.3)	2,299.4 (564.1)	2,327.4 (610.0)
Alcohol consumption [g/day] (mean, SD)^c^	10.9 (14.4)	10.4 (12.6)	12.3 (16.0)	4.0 (5.7)	3.8 (5.7)	3.1 (4.0)
Working hours (n, %)^c^						
< 35 hours/week	196 (10.6)	230 (9.7)	10 (10.5)	734 (20.6)	442 (20.8)	35 (20.8)
>= 35 hours/week	1,521 (82.1)	1,978 (83.5)	74 (77.9)	2,287 (64.3)	1,412 (66.5)	91 (54.2)
Unemployed	135 (7.3)	160 (6.8)	11 (11.6)	534 (15.0)	269 (12.7)	42 (25.0)
Chronic disease at baseline (n, %)						
Cardiovascular	128 (6.8)	148 (6.2)	9 (9.4)	100 (2.8)	73 (3.4)	3 (1.8)
Cancer	53 (2.8)	65 (2.7)	6 (6.3)	115 (3.2)	87 (4.1)	3 (1.8)

Standard deviation (SD), ^a ^Statistical significant difference in both sexes; ^b ^Statistical significant difference for men; ^c ^Statistical significant difference for women. The means differences were compared using Analysis of Variance and Tukey's test.

Among women, those who overestimated their body size showed a higher proportion of underweight, and more of them were unmarried in comparison with those who underestimated or correctly perceived their body size. The women who overestimated their body size also had lower average age, lower alcohol consumption, and a greater proportion of unemployment than those who underestimated or correctly perceived their body size.

We show in table 3 the results of the logistic regression models fitted to evaluate the association between body image disturbance and the incidence of depression. This relationship was not statistically significant in either sex. After additionally adjusting for the occurrence of pregnancy or the incidence of cardiovascular disease and cancer during follow-up, the association between body image disturbance and the incidence of depression remained without statistical significance.

**Table 3:** Association between body image disturbance and incidence of depression^a ^in participants of the SUN study according to sex.

	**Sex**					
	
	**Male**			**Female**		
	
	**Body image disturbance**			**Body image disturbance**		
	
	**None**	**Underestimated**	**Overestimated**	**None**	**Underestimated**	**Overestimated**
Cases	78	83	1	218	108	10
Total	1875	2397	96	3604	2145	169
OR (95% CI)^b^	1 (ref.)	0.83 (0.60 – 1.13)	0.24 (0.03 – 1.78)	1 (ref.)	0.81 (0.64 – 1.02)	1.02 (0.53 – 1.97)
OR (95% CI)^c^	1 (ref.)	0.83 (0.60 – 1.15)	0.23 (0.03 – 1.70)	1 (ref.)	0.84 (0.66 – 1.07)	0.81 (0.39 – 1.69)
OR (95% CI)^d^	1 (ref.)	0.83 (0.60 – 1.16)	0.21 (0.03 – 1.55)	1 (ref.)	0.85 (0.67 – 1.08)	0.82 (0.40 – 1.70)

Odds Ratio (OR); 95% Confidence Interval (95% CI); ^a ^Depression was defined as a physician diagnosis or the use of antidepressants drugs; ^b ^Odds Ratio of the association between body image disturbance and incidence of depression adjusted for age; ^c ^Odds Ratio of the association between body image disturbance and incidence of depression adjusted for age, weight change in the past five years (none, lost, gain), marital status (unmarried, married, other), smoking (never, current smoker, past smoker), physical activity (METs-h/week), energy intake (Kcal/day), alcohol consumption (g/day), working hours (< 35 hours/week, >= 35 hours/week, unemployed), chronic diseases (cardiovascular diseases and cancer) at baseline; ^d ^Odds Ratio of the association between body image disturbance and incidence of depression adjusted for the same variables in (c) plus pregnancy and chronic diseases (cardiovascular diseases and cancer) during follow-up.

Table 4 shows the results after restricting the case definition to the medical diagnosis of depression, eliminating the participants who only reported use of antidepressants during follow-up. Among women, underestimation of body size was inversely associated with the incidence of depression after adjusting for age (OR: 0.72, 95% CI: 0.54 – 0.95), but this relationship was not maintained after adjusting for other possible confounding variables.

**Table 4:** Association between body image disturbance and incidence of depression^a ^in participants of the SUN study according to sex.

	**Sex**					
	
	**Male**			**Female**		
	
	**Body image disturbance**			**Body image disturbance**		
	
	**None**	**Underestimated**	**Overestimated**	**None**	**Underestimated**	**Overestimated**
Cases	43	52	0	163	71	9
Total	1840	2366	95	3549	2108	168
OR (95% CI)^b^	1 (ref.)	0.94 (0.62 – 1.41)	e	1 (ref.)	0.72 (0.54 – 0.95)	1.20 (0.60 – 2.39)
OR (95% CI)^c^	1 (ref.)	0.92 (0.60 – 1.39)	e	1 (ref.)	0.76 (0.57 – 1.01)	0.95 (0.43 – 2.06)
OR (95% CI)^d^	1 (ref.)	0.92 (0.60 – 1.39)	e	1 (ref.)	0.77 (0.57 – 1.02)	0.96 (0.44 – 2.09)

Odds Ratio (OR); 95% Confidence Interval (CI 95%);^a ^Depression was defined only as a physician diagnosis, excluding those participants who reported use of antidepressant drugs, n = 160; ^b ^Odds Ratio of the association between body image disturbance and depression adjusted for age; ^c ^Odds Ratio of the association between body image disturbance and depression adjusted for age, body mass index, weight change in the past five years (none, lost, gain), marital status (unmarried, married, other), smoking (never, current smoker, past smoker), physical activity (METs-h/week), energy intake (Kcal/day), alcohol consumption (g/day), working hours (< 35 hours/week, >= 35 hours/week, unemployed), chronic diseases (cardiovascular diseases and cancer) at baseline; ^d ^Odds Ratio of the association between body image disturbance and incidence of depression adjusted for the same variables in (c) plus pregnancy and chronic diseases (cardiovascular diseases and cancer) during follow-up; ^e ^Odds Ratio could not be calculated.

**Figure 2:**
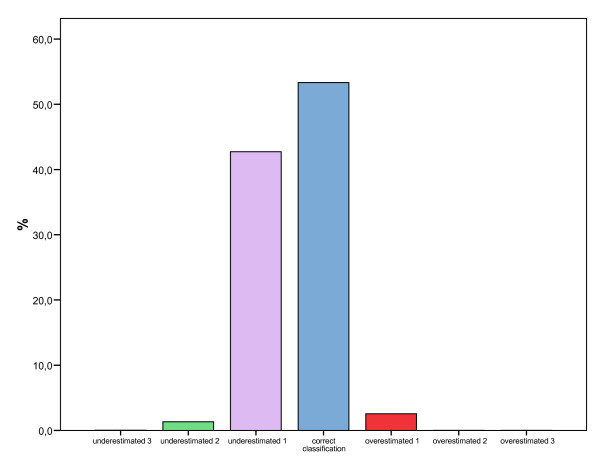
**Body image disturbance in participants of the SUN study according to sex**. Underestimated 3 = the person who underestimated 3 silhouettes below his/her body mass index; Underestimated 2 = the person who underestimated 2 silhouettes below his/her body mass index; Underestimated 1 = the person who underestimated 1 silhouettes below his/her body mass index; Correct classification = the person who assess correctly his/her body image; Overestimated 1 = the person who overestimated 1 silhouettes above his/her body mass index; Overestimated 2 = the person who overestimated 2 silhouettes above his/her body mass index; Overestimated 3 = the person who overestimated 3 silhouettes above his/her body mass index.

In figure 2 the categories of body image disturbance in the participants of this study are shown. The proportion of participants who correctly perceived their body size was high (53.3%) and gross misperception was seldom found, with most cases selecting only one silhouette below (42.7%) or above (2.6%) their actual BMI.

## Discussion

The results of our study do not support any association between body image disturbance and incidence of depression. However, our study was not informative for males who overestimated their body size, because we found a very small proportion of males in that category and a single case of incident depression among them.

This is in contrast to some results observed by other researchers who examined this topic [8,10,16,17]. However, those studies had a cross sectional design, which does not assure the temporal sequence of the association investigated. This problem is avoided in our work which has had a longitudinal design.

Other studies have shown that depression can lead to body image disturbance [12,13,15]. However, the temporal sequence is a potential major limitation of those studies as well, as they also used a cross sectional design.

We have no knowledge of any previous prospective study with a large sample and follow-up period that has evaluated the relationship between body image disturbance and depression among adults. One study conducted with ninety-seven patients with binge eating disorders has reported that body image disturbance is a predictive factor of depression [29].

One possible explanation for the lack of association between body image disturbance and incidence of depression in our adult population is the high educational level amongst our studied population. This characteristic may contribute to more accurate self-report of perceived body size. More than 50.0% of the participants correctly estimated their body size and the misperception was mostly limited to only one silhouette below the actual BMI, which can be considered as acceptable. Studies done by our group have shown that participants of this cohort have also correctly self-reported diagnoses for other important characteristics [23,28,30,31].

The lack of association between body image disturbance and incidence of depression in our study could also be explained because we considered the difference between BMI and the silhouettes proposed by Stunkard et al. (1983) [32] as a proxy of body image disturbance, which does not accurately evaluate this outcome. Other studies applied questionnaires to measure the degree of contentment with physical appearance, such as the Body Satisfaction Scale [33], the Body Image Avoidance Questionnaire [34], the Selves Questionnaire [35] and the Multidimensional Body Self-Questionnaire [36]. Nevertheless, a major concern in the assessment of body image has been the apparent failure to recognize the complexity of the body image construct. There have been important advancements in the development of standardized body image measurement tools in recent years. However, it would be premature to assume that any of these methods assesses body image in its entirety [37]. It has been proposed that the body image disturbance is not due to any perceptual deficit, but is based on cognitive-evaluative dissatisfaction [38]. Furthermore, some authors have used body image disturbance and body image dissatisfaction as synonyms and these terms do not have the same meaning [29].

Other limitations of this study were: (1) Depression was defined using the question, "Have you ever been diagnosed with depression by a health professional?" However, this question has been validated with this cohort by our group in previous research. In that study a self-reported physician-made diagnosis of depression was validated in our cohort using the Structured Clinical Interview for DSM-IV (SCID-I) applied by blinded experienced psychiatrists as the Gold Standard (submitted article). Other studies applied questionnaires for diagnosing depressive symptoms, particularly the Beck Depression Inventory [39]; (2) Unfortunately, we did not asses variables such as family history of depression or adverse life events which might impact on the incidence of depression [40-42]; (3) The differences between the participants and non-participants of this study could also influence our results.

Despite not being the initial objective of our study, it is important to highlight some secondary results that we observed. Men who underestimated their body size were those with higher rates of overweight and obesity. Among women, those who overestimated their body size had higher rates of underweight. These findings are consistent with the theory already presented in the introduction of this article which asserts that the aesthetic standards typical of Western cultures can influence body image disturbance [8,9]. Women want to lose weight, despite leanness, a fact which may contribute to the occurrence of anorexia and bulimia [43,44]. Men wish to gain muscle mass, despite having anthropometric standards within the normal range, which is contributing to the occurrence of muscle dysmorphia [45].

## Conclusion

We conclude that no association was found between body image disturbance and the incidence of depression in participants of the SUN study. Other longitudinal studies among adults should be conducted to confirm our results.

## Competing interests

The authors declare that they have no competing interests.

## Authors' contributions

AMP carried out the statistical analysis, drafted the manuscript and gave final approval of the version to be published.

ASV was involved in drafting the manuscript, revised it critically for important intellectual content and gave final approval of the version to be published.

MBR made substantial contributions to analysis and interpretation of data, was involved in drafting the manuscript, revised it critically for important intellectual content and gave final approval of the version to be published.

CNL was involved in drafting the manuscript, revised it critically for important intellectual content and gave final approval of the version to be published.

MAGM made substantial contributions to conception and design, acquisition of data, analysis and interpretation of data; was involved in drafting the manuscript, revised it critically for important intellectual content and gave final approval of the version to be published.

## Pre-publication history

The pre-publication history for this paper can be accessed here:


